# Laparoscopic Cholecystectomy in the Elderly: An Experience at a Tertiary Care Hospital in Western Nepal

**DOI:** 10.1155/2017/8204578

**Published:** 2017-05-10

**Authors:** Tika Ram Bhandari, Sudha Shahi, Rajeev Bhandari, Rajesh Poudel

**Affiliations:** ^1^Department of General Surgery, Universal College of Medical Sciences, Bhairahawa 32900, Nepal; ^2^Department of ENT, National Academy of Medical Sciences, Kathmandu 44600, Nepal

## Abstract

**Background:**

The incidence of gallstone increases with increasing age. No studies have been reported in the elderly population with laparoscopic cholecystectomy from developing nations. The aim of this study was to compare perioperative outcomes of laparoscopic cholecystectomy between the elderly (≥60 years old) and the young (<60 years old).

**Methods:**

From July 2015 to June 2016, a retrospective review of medical records of 78 elderly patients (≥60 years old) and 164 young patients (<60 years old) who underwent laparoscopic cholecystectomy was done. The patients' demographics and perioperative outcomes were analyzed.

**Results:**

Median ages were 65 years (range: 60–80) and 45 years (range: 21–59) for the elderly group and the young group. The majority of patients were female (62.8 and 72%). There were no significant differences in the conversion rate (9 and 7.9%, *P* = 0.78), postoperative complications (17.9 and 14.6%, *P* = 0.50), and length of stay in the hospital (4 days for both groups, *P* = 0.35) between the two groups. There was no mortality in either of the groups.

**Conclusion:**

Our results of laparoscopic cholecystectomy in elderly patients are comparable with those in young patients. Therefore, laparoscopic cholecystectomy is safe even in the elderly population.

## 1. Introduction

The incidence of gallstone disease in the elderly population ranges from 14% to 27% according to various studies [1]. Laparoscopic cholecystectomy is recognized as the gold standard for the surgical management of gallstone diseases. Surgery for cholelithiasis is more common in elderly patients as the incidence of gallstones increases with age (13–50%) [2]. Age is one of the critical factors affecting the mortality and morbidity rates after cholecystectomy [3]. The use of a laparoscopic procedure in elderly patients may cause problems because comorbid conditions are very common with advanced age and may increase the postoperative complications and the frequency of conversion to open surgery [4]. It has been reported that laparoscopic cholecystectomy in the elderly has comparable safety and efficacy to those in younger populations [5]. However, no studies have been reported in developing countries on the elderly yet. The aim of this study is to evaluate the outcome of LC in the elderly as compared to young populations in developing countries like ours with limited resources.

## 2. Materials and Methods

This is a retrospective review of the medical records of all patients who underwent laparoscopic cholecystectomy at the Universal College of Medical Sciences teaching hospital, Bhairahawa, situated in Western Nepal, from July 2015 to June 2016. Patients were divided into two groups based on age: elderly (≥60 years, *n* = 78) and young (<60 years, *n* = 164). Patients' demographics and operative details were analyzed. All patients were evaluated with abdominal ultrasonography and baseline investigations required for surgery. Magnetic resonance cholangiopancreatography (MRCP) was performed only in selected patients with suspected choledocholithiasis or with dilated biliary duct in ultrasonography. Preoperative endoscopic retrograde cholangiopancreatography (ERCP) was utilized in patients with common bile duct stones.

The timing of laparoscopic cholecystectomy in patients presenting with acute phase was after the resolution of symptoms. Most of these patients were treated conservatively, discharged, and readmitted for elective surgery. Perioperative antibiotic prophylaxis with second-generation cephalosporin was given to all patients. Laparoscopic cholecystectomy was performed by the standard four-port technique. We used the open technique to introduce a subumbilical cannula. We had monopolar electrocautery to dissect the gallbladder from the liver bed. We regularly put titanium clips for cystic duct and cystic artery ligations. Routine intraoperative cholangiography was not performed. A closed suction drain was put in selected cases according to need.

Perioperative data including conversion rate, duration of surgery, postoperative complications, resumption of normal diet, and length of hospital stay were recorded. Bilious drain with elevated bilirubin in the drain was defined as biliary leakage. Surgical site infection was defined according to surgical site infection (SSI) guidelines. Intra-abdominal abscess (IAA) was defined as culture positive purulent collection. The length of hospital stay was considered as the period from the first postoperative day until discharge from the hospital. All continuous variables were expressed as median or mean and standard deviation which compared with independent *t*-test or Mann–Whitney test as feasible, according to the type of distribution. Chi-square test or Fisher's exact test was used for categorical values as appropriate. Statistical software SPSS version 22.0 (Statistical Package for the Social Sciences) was used for statistical analysis. A *P* value < 0.05 was considered statistically significant.

## 3. Results

A total of 242 patients undergoing laparoscopic cholecystectomy during the study period comprised 78 elderly patients and 164 young patients. Median ages were 65 years (range: 60–80) for the elderly group and 45 years (range: 21–59) for the young group. The majority of patients were female (62.8 and 72%) in respective groups. All patients had symptoms of gallstone disease like fatty meal intolerance, belching and bloating, biliary colic, epigastric pain, and right upper quadrant pain with radiation to the infrascapular region. Patients' preoperative data are shown in Table 1. There were no significant differences in different preoperative variables except comorbidity (*P* < 0.05), when we compared these variables in elderly and young patients.

We did not find significant differences in indications for laparoscopic cholecystectomy between the groups, with the most frequent diagnosis of biliary colic for both groups (*P* = 0.87) as shown in Table 2.

We observed 9% conversion to open surgery in elderly patients and 7.9% in young patients (*P* = 0.78) as shown in Table 3. The most common cause for conversion was failure to adequately visualize the biliary tract anatomy and Calot's triangle due to intense fibrosis followed by uncontrolled intraoperative bleeding. The mean operative time was not different between groups, with an average of 50 (range: 30–90 min) minutes for the elderly and 50 (range: 40–100 min) minutes for the young group (*P* = 0.82) as shown in Table 3.

No significant difference in complication rate for elderly and young patients (17.9 and 14.6%, *P* = 0.508) was observed. The most common complications were lower respiratory tract infection in the elderly and superficial thrombophlebitis in the young group (Table 3). Although there was no bile duct injury observed in our study, 1 patient in the elderly group and 3 patients in the young group had minor bile leak which was managed conservatively with ultrasonographic guidance tube drainage. One case of primary hemorrhage occurred in the elderly group and 2 occurred in the young group which was due to slippage of cystic artery ligature and was managed with reoperation and ligation of the bleeding cystic artery. There was no mortality in either of the groups. The median time to resumption of normal diet was not different between the groups, with an average of 1 day (1-2 days) for both groups (*P* = 0.47). The median length of hospital stay was also similar between the groups, with an average of 4 days (1–18 days) for the elderly and 4 days (2–18 days) for the young group (*P* = 0.35) as shown in Table 3. In logistic regression analysis, on multivariate analysis, out of independent variables age (≥60 years), sex (male), presence of comorbidity, and duration of surgery (>60 min), only presence of comorbidity was associated with an increased risk of postoperative complications (odds ratio: 4.03, 95% confidence interval; [0.117–0.529], *P* < 0.05).

## 4. Discussion

Life expectancy of people has increased even in the developing countries over few decades, perhaps due to improving levels of primary prevention and advancement of medical care. For the developing nations, the World Health Organization (WHO) has defined the elderly as people aged 60 or older and 65 and over for developed nations. As we are based in a developing nation, we included ≥60 years as the elderly population in our study. As life expectancy of the population is rising, the surgical needs of aging patients are increasing. The increasing age of the people has led to a rising prevalence of gallstones; therefore, cholecystectomy is a common surgical procedure in elderly patients [2]. As compared with open surgery, laparoscopic cholecystectomy (LC) has the benefits of less pain, shorter length of stay in the hospital, and early return to work [6–9]. Age should not be considered as a contraindication for LC, although the laparoscopy surgery was initially reserved for low-risk patients [10].

When we compared outcomes of laparoscopic cholecystectomy in the elderly and young population, we did not find statistical differences in the mean operative time, the rate of complications, and conversion to open cholecystectomy between the elderly and young groups (*P* > 0.05). There was also no significant difference in the median time to resumption of normal diet and length of hospital stay between the two groups. There was no mortality in either group, although the reported morbidity and mortality for laparoscopic cholecystectomy in the elderly patients vary from 5% to 15% and 0% to 1%, respectively [11–14].

The majority of patients in this study underwent laparoscopic cholecystectomy for recurrent biliary colic and chronic symptoms which are similar to the ones reported [4, 15]. The mean operative time of 50 minutes (range: 30–90 min) in the elderly in our study is comparable to that in other studies [16]. We had 9% conversion to open cholecystectomy in elderly patients and 7.9% in young patients (*P* = 0.78). Our conversion rate in the elderly is lower than that previously reported [17–20] which may be due to the selection of patients. Many studies mention that aging patients are more likely to have chronic symptoms of gallbladder disease. Most of these patients suffer from repeated inflammation resulting in a contracted gallbladder with dense adhesions to surrounding structures, rendering laparoscopic surgery difficult [21, 22].

We did not observe a significant difference in complications between the groups (17.9 and 14.6%, *P* = 0.508). The complications (17.9%) found in elderly patients are lower than those reported in other studies [4, 23]. However, the rate of these complications is higher than that reported by Rubert et al. [24]. There was no significant difference in terms of resumption of normal diet and length of stay in the hospital postoperatively for elderly and young patients (*P* > 0.05). The postoperative stay of 4 days (range: 1–18 days) in the elderly is comparable to that in other studies [25, 26].

We also analyzed different factors that affect postoperative outcomes. The presence of comorbidity was an independent risk factor for postoperative complications which is similar to what is reported by other studies [12]. We did not find any correlation between the age of the patient and the postoperative morbidity. This is in accordance with other reports which conclude that the surgery should not be denied solely on the basis of age [8, 16]. However, Vivek et al. in their study reported that aging is an independent risk factor associated with postoperative morbidity with laparoscopic cholecystectomy [27].

Galizia et al. in their study described that significant physiologic changes due to insufflation of CO_2_ for abdominal distention can occur during the laparoscopic procedure, mainly in aged patients with concomitant comorbid diseases. Complex hemodynamic change that occurs during laparoscopy due to pneumoperitoneum frequently increases right and left side filling pressure, disrupts respiratory mechanisms, and causes respiratory acidosis [28]. Although our elderly patients had additional comorbid diseases, we did not find intraoperative complications during laparoscopic cholecystectomy. It has been mentioned that pneumoperitoneum also raises cardiac sympathetic activity and increases the chance of fatal arrhythmias. Therefore, possibly in the future, advanced gasless laparoscopy may be beneficial for high-risk elderly patients even in developing countries like ours. Our experience of conducting laparoscopy with intra-abdominal pressure, up to 10–12 mmHg, prevents hazards in high-risk patients. A study reported that cardiopulmonary alterations associated with laparoscopic cholecystectomy can be also minimized by a wall lift procedure during laparoscopy [29].

Shortage of skilled staff and training opportunities, repeated use of disposable operative instruments, and inadequacy of means to maintain equipment are common challenging tasks in limited resource settings. In addition to that, people frequently have poor access to health services because of poverty, poor transport facilities, and long distances in rural situations. Generally, our elderly patients doubt the safety and efficacy of the laparoscopic technique and cannot remark the exact benefit because of the lack of education, poor health knowledge, and social beliefs.

Despite all, we believe that this is the first study in the developing world suggesting that laparoscopic cholecystectomy is safe in the elderly patients. Our results of laparoscopic cholecystectomy in elderly (aged 60 or above) patients are comparable with those previously reported studies. The limitations of our study were the small elderly study population and the retrospective and single centered design. We could not include some data like patients' BMI, which was not mentioned in hospital records. We also acknowledge our limitation of not being able to compare outcomes of laparoscopic cholecystectomy with open cholecystectomy in the elderly patients. Hence, in order to validate our findings, further appropriately designed prospective studies are recommended especially in developing countries with limited resources.

## 5. Conclusion

Our results of laparoscopic cholecystectomy in elderly (aged 60 or above) patients are comparable with young patients. Therefore, laparoscopic cholecystectomy is safe even in the elderly. Better results can be achieved even in low to medium volume centers with a dedicated team in developing countries with limited resources.

## Figures and Tables

**Table 1:** Preoperative data.

Variable	Elderly (*n* = 78)	Young (*n* = 164)	*P* value
Age (years)	65 (60–80)	45 (21–59)	0.001^*∗*^
Sex			0.151
Male	29 (37.2)	46 (28)	
Female	49 (62.8)	118 (72)	
ASA^*♣*^			0.091
I	62 (79.5)	117 (71.3)	
II	13 (16.7)	45 (27.4)	
III	3 (3.8)	2 (1.2)	
IV	0 (0)	0 (0)	
Comorbidity, %	28 (35.9)	27 (16.5)	0.001^*∗*^
Cardiovascular disease	5 (6.4)	5 (3.0)	
Diabetes mellitus	9 (11.5)	13 (7.9)	
Respiratory diseases	4 (5.1)	2 (1.2)	
Renal disease	2 (2.4)	2 (1.2)	
Hypertension	7 (9.0)	6 (3.7)	
Neurological problems	1 (1.3)	1 (0.6)	

Categorical variables are presented as *n* (%). Continuous variables are presented as median (interquartile range). ^*♣*^ASA: American Society of Anesthesiology; ^*∗*^*P* value < 0.05.

**Table 2:** Indications for laparoscopic cholecystectomy.

Clinical and pathological diagnosis	Elderly (*n* = 78)	Young (*n* = 164)	*P* ^*∗*^

Biliary colic	35 (44.9)	91 (55.5)	0.874
Chronic calculous cholecystitis	20 (25.6)	40 (24.4)	
Acute calculous cholecystitis	5 (6.4)	8 (4.9)	
Mucocele of gallbladder	6 (7.7)	7 (4.3)	
Empyema gallbladder	4 (5.1)	6 (3.7)	
Acute biliary pancreatitis	3 (3.8)	5 (3.0)	
Polyp with gallstone	2 (2.6)	4 (2.4)	
Carcinoma gallbladder	2 (2.6)	2 (1.2)	
Acute acalculous cholecystitis	1 (1.3)	1 (0.6)	

^*∗*^ *P* value significant if <0.05.

**Table 3:** Intraoperative and postoperative data.

Variable	Elderly (*n* = 78)	Young (*n* = 164)	*P* ^*∗*^
Operative time (min)	50 (30–90)	50 (40–100)	0.82
Operative time (>60 min)	38 (48.7)	69 (42.1)	0.33
Conversion to open cholecystectomy	7 (9.0)	13 (7.9)	0.78
Complications, %	14 (17.9)	24 (14.6)	0.50
LRTI^¶^	4 (5.1)	5 (3.0)	
UTI^†^	3 (3.8)	3 (1.8)	
Superficial thrombophlebitis	3 (3.8)	7 (4.3)	
Intra-abdominal abscess	1 (1.3)	2 (1.2)	
Hemorrhage	1 (1.3)	2 (1.2)	
Bile leak	1 (1.3)	3 (1.8)	
SSI^*♦*^	1 (1.3)	2 (1.2)	
Time to resumption of normal diet (days)	1 (1-2)	1 (1-2)	0.47
Length of hospital stay (days)	4 (1–18)	4 (2–18)	0.35

Categorical variables are presented as *n* (%). Continuous variables are presented as median (interquartile range). ^¶^LRTI: lower respiratory tract infection; ^†^UTI: urinary tract infection; ^*♦*^SSI: surgical site infection; ^*∗*^*P* value significant if <0.05.
